# The Role of Th17/Treg Axis in Retinal Pathology Associated with Diabetes and Treatment Options

**DOI:** 10.3390/biology14030275

**Published:** 2025-03-07

**Authors:** Michel-Edwar Mickael, Norwin Kubick, Kreshnik Miftari, Jarosław Olav Horbańczuk, Atanas G. Atanasov, Korona Binçe, Piotr Religa, Agnieszka Kamińska, Mariusz Sacharczuk, Michał Ławiński

**Affiliations:** 1 Institute of Genetics and Animal Biotechnology, Polish Academy of Sciences, Postępu 36A, 05-552 Jastrzebiec, Poland; j.horbanczuk@igbzpan.pl (J.O.H.); a.atanasov@igbzpan.pl (A.G.A.); m.sacharczuk@igbzpan.pl (M.S.); 2 Department of Biology, Institute of Plant Science and Microbiology, University of Hamburg, Ohnhorststr. 18, 22609 Hamburg, Germany; kubick.norwin@googlemail.com; 3 Faculty of Medicine, University of Prishtina, Str. “George Bush”, No. 31, 10 000 Prishtine, Kosovo; kreshnik.miftari@student.uni-pr.edu (K.M.); korona.bince@student.uni-pr.edu (K.B.); 4 Ludwig Boltzmann Institute Digital Health and Patient Safety, Medical University of Vienna, 1090 Vienna, Austria; 5 Department of Laboratory Medicine, Division of Pathology, Karolinska Institute, SE-141 86 Stockholm, Sweden; piotr.religa@ki.se; 6 Faculty of Medicine, Collegium Medicum, Cardinal Stefan Wyszyński University in Warsaw, 01-938 Warsaw, Poland; agnieszka.kaminska73@wp.pl; 7 Department of Pharmacodynamics, Faculty of Pharmacy, Medical University of Warsaw, Banacha 1B, 02-091 Warsaw, Poland; 8 Department of General Surgery, Gastroenterology and Oncology, Medical University of Warsaw, 02-091 Warsaw, Poland; m.lawinski@igbzpan.pl

**Keywords:** diabetic retinopathy (DR), blood–retinal barrier (BRB), Th17 cells, Treg cells, infiltration

## Abstract

Diabetic retinopathy (DR) is a major complication of diabetes, leading to vision impairment and blindness. The pathogenesis of DR involves multiple factors, including hyperglycemia-induced vascular damage, hypertension, obesity, anemia, immune dysregulation, and disruption of the blood–retinal barrier (BRB). Th17 and Treg cells, two types of CD4+ T cells, play opposing roles in inflammation. Th17 cells are pro-inflammatory, producing cytokines such as IL-17A, while Treg cells help suppress immune responses and promote anti-inflammatory effects. Recent studies highlight the importance of the Th17/Treg balance in retinal inflammation and disease progression in DR. Our literature review reveals an imbalance in DR, with increased Th17 activity and reduced Treg function. This shift creates a pro-inflammatory environment in the retina, worsening vascular leakage, neovascularization, and vision loss. The limited infiltration of Treg cells suggests that Th17 cells may uniquely infiltrate the retina by overwhelming or outnumbering Tregs or increasing the expression of recruiting chemokines, rather than only taking advantage of a damaged BRB. Therapeutic strategies, such as neutralizing IL-17A and enhancing Treg function with compounds like IL-35 or curcumin, may reduce inflammation and retinal damage. Restoring the balance between Th17 and Treg cells could provide new approaches for treating DR by controlling inflammation and preventing further retinal damage.

## Simple Summary

Diabetic retinopathy (DR), a major complication of diabetes, damages retinal blood vessels, disrupts the blood–retinal barrier (BRB), and impairs vision. Th17 and Treg cells, subsets of CD4+ T cells, play opposing roles: Th17 cells promote inflammation and tissue damage, while Treg cells suppress inflammation and maintain retinal homeostasis. We propose that Th17 cells infiltrate the retina more efficiently through the compromised BRB in DR, while Treg cells show reduced infiltration, creating a pro-inflammatory environment that worsens vascular leakage, neovascularization, and vision loss. Modulating the Th17/Treg balance through cytokine modulation or Treg-based therapies may restore immune homeostasis and alleviate DR symptoms. Future research should explore mechanisms behind this differential infiltration to develop targeted treatments.

## 1. Introduction

DR is a microvascular complication of diabetes mellitus which is characterized by progressive damage to the blood vessels in the retina [1,2]. It affects individuals across all age groups, with a prevalence of 34.6% (approximately 93 million) among adults aged 40 and older [3]. DR is classified into non-proliferative diabetic retinopathy (NPDR) and proliferative diabetic retinopathy (PDR) based on the modified Airlie House Classification, as outlined in the Early Treatment Diabetic Retinopathy Study (ETDRS) [4]. The earliest morphological indicator of non-proliferative diabetic retinopathy (NPDR) is the appearance of microaneurysms, where capillary walls bulge outward, which can be observed through ophthalmoscopy, often accompanied by blot hemorrhages [5,6]. Additional NPDR features include altered retinal blood flow, increased vascular permeability, basement membrane thickening, pericyte loss, and the development of acellular capillaries [7]. As ischemia worsens, NPDR may progress to proliferative diabetic retinopathy (PDR), which is marked by abnormal retinal neovascularization, vitreous hemorrhages, new blood vessels in the vitreous, and retinal traction detachments, potentially leading to blindness [8,9].

Current treatments are not able to cure DR, but they aim to slow its progression and prevent vision loss [10]. Laser therapy, including focal laser treatment to target leaking blood vessels and panretinal photocoagulation to reduce abnormal blood vessel growth, is commonly used [11,12]. However, its disadvantage is that it can cause peripheral vision loss and potential scarring of the retina [13]. Targeting molecules responsible for mediating neovascularization is a viable therapeutic approach. One such molecule is vascular endothelial growth factor (VEGF), a potent angiogenic factor that plays a crucial role in vascular endothelial cell growth. Anti-VEGF injections, such as bevacizumab, ranibizumab, and aflibercept, help reduce abnormal blood vessel formation and fluid accumulation in the retina. These treatments provide significant benefits in controlling edema and managing diseases characterized by pathological angiogenesis [14]. The downside is that repeated injections are required, which can be costly and invasive, with potential side effects like increased eye pressure or retinal detachment [15]. Steroid injections can be effective in reducing inflammation and treating macular edema, but long-term use may increase the risk of cataracts, glaucoma, and further retinal damage. In advanced cases, vitrectomy surgery is used to remove blood or scar tissue in the vitreous humor, but it carries risks such as retinal detachment, cataract formation, and infection [16]. Despite these options, managing diabetes through blood sugar, blood pressure, and cholesterol control remains the cornerstone of preventing further retinal damage, but it does not directly address the existing retinal changes caused by DR [2,17,18,19].

Chronic hyperglycemia is strongly associated with low-grade inflammation at various stages of DR in both animal models and human patients [20]. This inflammation is driven by multiple mechanisms [21,22]. For example, chronic hyperglycemia has been linked to leukostasis, characterized by white blood cell accumulation and blood flow obstruction [23,24]. Increased leukostasis has been spatially correlated with endothelial damage and BRB impairment in diabetic rats [25,26]. Leukocyte–endothelium adhesion, mediated by adhesion molecules, is a key factor in leukostasis during diabetes [27,28]. Elevated leukocyte adhesion and upregulation of β2-integrins, including CD11a, CD11b, and CD18, have been observed in diabetic rats and patients [29]. Chemokines also play a crucial role in leukocyte attraction and activation during DR, with elevated levels of MCP-1, MIP-1α, and MIP-1β reported in diabetic patients [30]. Inflammatory cytokines such as TNF-α, IL-6, IL-8, and IL-1β are significantly upregulated in DR, correlating with disease severity [31,32]. Retinal glial cell dysfunction is another contributing factor. Under hyperglycemic conditions, activated microglia secrete increased levels of TNF-α, IL-6, MCP-1, and VEGF [33]. Müller cells and astrocytes further amplify inflammation by producing pro-inflammatory cytokines [34,35,36]. Emerging evidence highlights the critical role of adaptive immunity in DR progression [37,38]. In particular, hyperglycemia-induced metabolic changes and oxidative stress drive a pro-inflammatory state, exacerbating retinal damage. Elevated IL-17 levels in the vitreous humor of patients with PDR underscore the role of Th17-mediated inflammation, whereas Tregs appear to have a protective effect by modulating immune responses and reducing inflammation. Single-cell analysis of PDR patients revealed that T cells, particularly CD4+, CD8+, and Tregs, dominate in the vitreous, whereas they are less abundant in peripheral blood [39]. The analysis also showed activation of memory T cells and specific ligand–receptor interactions unique to the vitreous, with neutrophils being virtually absent. However, the precise role of the Th17/Treg balance in DR development remains not fully understood. This review evaluates the role of Th17 and Treg cells in DR pathogenesis. Potential therapeutic strategies to restore Th17/Treg immune balance are also discussed.

## 2. The Th17/Treg Axis

**Figure 1:**
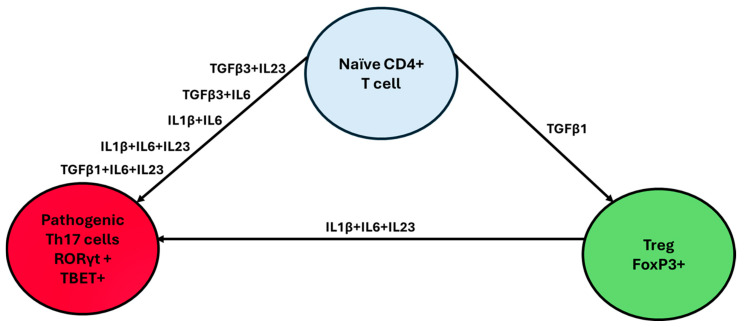
Th17/Treg axis differentiation. The microenvironment chemokines that dominate the milieu of naïve CD4+ T cells play a crucial role in Th17/Treg differentiation. Interestingly, the presence of TGF-β1 alone promotes Treg differentiation, while TGF-β1 combined with pro-inflammatory cytokines such as IL-1β, IL-6, or IL-23 drives differentiation toward the Th17 lineage.

The Th17/Treg axis plays a fundamental role in autoimmunity. Th17 and Treg cells both differentiate from CD4+ T cells and share a large portion of their transcriptome; however, they differ significantly in their functions. The Th17 cells represent a major lineage of CD4+ T-cells that contribute to the maintenance of the first line of defense against pathogens at barrier surfaces, while conversely also playing a critical role in the development of autoimmune diseases [40,41]. Naïve CD4+ T cells can differentiate into Th17 cells depending on the cytokine combinations present in their microenvironment. These combinations include TGFB3+IL23, TGFB1+IL6+IL23, TGFB3+IL6, TGFB1+IL6, IL21+TGFB1, IL1B+IL6+IL23, IL1B+IL6, and IL21+TGFB1, with differentiation occurring through a pathway mediated by the IL21 receptor (Figure 1) [42]. These cytokines, through activating TGFβ and SMAD, initiate the phosphorylation of Signal Transducer and Activator of Transcription 3 (STAT3), ultimately leading to the activation of RORγt, which is the main transcriptional regulator of Th17 cells, as well as other key transcription factors such as RORα, BATF, and IRF4 [43]. These transcription factors drive the production of three main cytokines, namely IL17A, IL17F, and IL22 [44,45,46]. These cytokines, in turn, have been implicated in the stimulation of the secretion of pro-inflammatory molecules, further participating in immunity against bacterial or fungal infections and in the pathogenesis of autoimmune or metabolic diseases such as multiple sclerosis, Alzheimer’s, rheumatoid arthritis, Parkinson’s, and IBD [47,48].

Regulatory T cells (CD25+CD4+ Tregs) play an integral role in maintaining homeostasis by exhibiting suppressive effects on Th1, Th17, and Th2 cells [33]. Natural Tregs (nTregs) are characterized by the expression of the transcription factor forkhead box P3 (Foxp3) in the thymus [49,50,51]. Induced Tregs develop in peripheral tissues (pTregs), where TGFβ activates FoxP3 through the SMAD-dependent pathway while simultaneously inhibiting STAT3 via an IL2R-STAT5 signaling pathway [52,53]. The significance of Tregs is exemplified by the spontaneous onset of autoimmune diseases observed in normal rodents upon depletion of CD25+CD4+ T cells [34]. Similarly, severe autoimmune conditions, allergies, and immunopathologies have been documented in both humans and rodents carrying mutations in the Foxp3 gene, further emphasizing the indispensable role of Tregs in preventing immune dysregulation [54]. The pathways and genes involved in immune regulation by T regulatory cells include both indirect and direct mechanisms. In the indirect pathway, genes such as CTLA4, CD28, CD80, CD86, LFAI-1, A20, CD40-CD40L, neuropilin-1, and LAG3 inhibit dendritic cell activity. The direct pathways involve the production of suppressive cytokines like TGFβ, IL10, and IL35, as well as cytokine consumption mediated by genes such as IL2, IL2Rα, IL2Rβ, and IL2Rγ. Apoptosis induction involves TRAIL, CD3, CD46, CD25, and BIM, while transcription factor regulation includes IRF4, GATA3, FoxP3, and RORγt [55]. Additionally, ATP and ADP regulation involves CD73, CD39, APRT, A2A receptors, and P2RY11, while cAMP/NFAT regulation involves IL4, ICER, PPRγ, p21SNFT, GITR, and CBLB [56]. Finally, calcium signaling is regulated by genes such as NFκB, PPP3CA, PPP3CB, PPP3CC, IKKα, IKKβ, IKKγ, IκB, IKBA, and p65 [57,58].

The Th17/Treg axis exhibits significant plasticity [47,59]. FoxP3+ Treg cells can be reprogrammed into Th17 cells under the influence of IL6. Exposure to IL6, IL123, and IL1B can transform Treg cells into pathogenic Th17 cells. Additionally, Treg cells may be reprogrammed into RORγt+FOXP3+IL17+ cells, such as Tr17. Conversely, Th17 cells can transition into anti-inflammatory Treg-like cells, such as Tr1, which are capable of reducing inflammation in the central nervous system (CNS). Intermediate stable phenotypes, such as RORγt+FoxP3+IL17− cells, have also been observed [60].

## 3. The Th17/Treg Axis in DR

Th17 cells may play a direct or indirect role in DR. Evidence from various studies suggests that Th17 cells can migrate from the bloodstream into intraocular tissues by crossing the retinal vascular endothelium [31,32,37,39,43]. Once in the retina, they contribute to inflammatory activity within the retinal pigment epithelium (RPE) and uvea [61,62,63]. Additionally, through the production of IL-17, Th17 cells may indirectly promote DR progression [43]. For instance, an examination of plasma from type 2 diabetes patients at different stages of DR progression using Luminex multiplex bead immunoassays revealed that IL-17 levels were significantly elevated in diabetic patients with DR symptoms [43]. In contrast, Chen et al. reported that the frequency of Th17 cells and IL17A levels in peripheral blood mononuclear cells (PBMCs) was significantly lower in patients with DR than in those without DR, and tended to decrease with increasing DR severity [64]. However, this hypothesis was challenged by findings showing upregulation of IL-17 in the aqueous humor of DR patients [31,65]. Further supporting the role of IL17, IL17A levels were significantly higher in the vitreous of PDR compared to those with non-diabetic retinopathy (non-DMR). This was confirmed by multiplex bead assays analyzing vitreous samples from 35 PDR eyes, showing its association with the detrimental effects of diabetes on the retina [66]. Additionally, a study of 64 participants found significantly higher concentrations of cytokines such as IL6, IL12, IL17A, and TNFα in DR patients compared to healthy controls, with the highest cytokine levels observed in the non-proliferative diabetic retinopathy (NPDR) group [67]. The role of IL17A in DR was further supported by Liu et al., who examined the correlation between serum IL17A levels and DR in elderly individuals with type 2 diabetes mellitus. Their study found that higher serum IL17A levels were significantly associated with more severe DR, with logistic regression analysis confirming a higher risk of both non-proliferative and proliferative diabetic retinopathy in individuals with elevated IL17A levels [68]. Moreover, IL17-RC, a component of the IL17A receptor complex, has been detected in retinal photoreceptors, retinal endothelial cells, and Müller glia, suggesting its involvement in the pathological processes of DR [69].

Direct evidence of a role of Th17 in the development of DR was further supported by the study of Manzo-Taguchi et al. In this study, the researchers investigated Th17-mediated immune responses in DR using a novel mouse model, Ins2Akita IFN-γ-deficient (Akita-GKO) mice, created by crossbreeding diabetic Ins2Akita mice with IFN-γ knockout (GKO) mice [70]. These mice exhibited enhanced differentiation and activation of Th17 cells due to the inhibition of Th1 responses. The study measured blood glucose levels, cytokine profiles, and retinal changes across Akita-GKO, Akita, GKO, and wild-type (WT) mice. The results revealed that Akita-GKO mice had significantly upregulated expression of the Th17 transcription factor ROR-γt and elevated proportions of IL-17 and IL-22-producing splenic CD4+ T cells compared to the other groups. In the retina, Akita-GKO mice showed increased mRNA expression of VEGF and ICAM-1, along with significant leukostasis, elevated VEGF protein levels, and vascular basement membrane thickening. Fluorescein angiography (FA) further demonstrated edematous changes and exudative lesions in Akita-GKO mice, which were not observed in Akita or GKO mice [70]. These findings suggest that Th17-mediated immune responses exacerbate both functional and morphological retinal changes in diabetic mice.

However, Th17 cells might not be the only source of IL17 in the retina. For example, retinal Müller cells (RMC), which are specialized glial cells essential for maintaining retinal homeostasis, play critical roles in BRB integrity, neuronal apoptosis, and glutamate metabolism [71]. Dysfunction and activation of Müller cells have been associated with retinal inflammation. A study by Qiu et al. showed that high glucose (HG) exposure induced the expression and secretion of IL17A and IL17AR in both primary Müller cell cultures and retinal tissue of diabetic mice. This indicates that Müller cells serve as both a target and a source of IL17A in DR [72].

An experimental study by Byrne et al. demonstrated that IL17A contributes to BRB dysfunction through activation of the JAK1 signaling pathway, both in vitro and in vivo. Furthermore, blocking JAK1 with Tofacitinib citrate, an anti-inflammatory drug, effectively prevented IL17A-induced macular edema and inflammation [73]. The pro-apoptotic effects of IL17A on retinal endothelial cells were also highlighted in a study using a streptozotocin (STZ)-induced murine model of type 2 diabetes mellitus. In *Il17a*^−/−^ mice, retinal capillary degeneration was significantly reduced. Mechanistically, the IL17A/IL17R/Act1 signaling cascade recruits the Fas-associated death domain (FADD), an adaptor protein involved in apoptosis. This interaction between Act1 and FADD activates caspases 3 and 8, leading to apoptosis of retinal endothelial cells [69,74].

IL17 action could take place through an epigenomic pathway. In one interesting study, diabetic alterations were induced in mice using streptozotocin (STZ), and retinal pigment epithelium (RPE) cells were subsequently purified for gene expression profiling [75]. KEGG pathway analysis identified the IL17 signaling pathway as the most significantly enriched and the sole inflammation-related pathway in the dataset. The researchers further demonstrated that IL17A induced the expression of targeted inflammatory genes in RPE cells, with this effect being enhanced under high-glucose conditions, indicating a synergistic interaction. Interestingly, high glucose levels did not influence the mRNA stability or IL17A signaling activity but instead increased histone acetylation on IL17A-targeted genes, amplifying their expression. These findings suggest that the interplay between IL17A and high glucose drives inflammation in RPE cells through an epigenomic mechanism [75].

Interestingly, cytokines responsible for mediating the differentiation of Th17 cells are upregulated in DR. For example, Chen et al. investigated the role of interleukin (IL)-6 trans-signaling in DR by measuring the concentrations of IL6, soluble IL6 receptor (sIL6R), and soluble gp130 (sgp130, an IL6 trans-signaling antagonist) in the serum and aqueous humor (AqH) of diabetic patients [76]. The study revealed significantly elevated levels of sgp130 in both the serum and AqH of DR patients compared to NDR patients and healthy controls. Additionally, higher concentrations of IL-6 and sIL-6R were observed in DR patients, with significant correlations between AqH sgp130, sIL-6R, and IL-6. Elevated levels of these markers were also associated with longer disease duration and higher metabolic indices, such as body mass index, plasma glucose, and HbA1c. These findings suggest that IL6 trans-signaling plays a potential role in the pathophysiology of DR. Similar results were observed in different populations and animal models exhibiting DR symptoms [77,78,79,80].

Several studies have indicated that Treg cells in type II diabetes and diabetic retinopathy patients are reduced in number and capacity [81,82,83]. Treg cells are beneficial in protecting against diabetic retinopathy in diabetes. In an interesting study, Llorián-Salvador et al. expanded Treg numbers through the injection of IL-2 in an established mouse model for diabetes, known as *db*/*db*. Immunohistochemistry was utilized to assess retinal neurons, glia, and vascular permeability, using markers such as Cone-Arrestin, PKCα, synaptophysin, ChAT, TH, GFAP, Iba-1, calbindin, Brn3a, RBPMS, isolectin B4, and albumin. Additionally, retinal VEGF levels were measured, and NLRP3, Casp1, p20, and IL-18 were analyzed in retinal homogenates. The results showed a significant downregulation of inflammation markers, suggesting a potential role for Tregs in providing protection against retinal neurodegeneration in type 2 diabetes [84]. However, interestingly, Treg expansion was not associated with improvements in the diabetic state of the mice. This could be due to the specific nature of the *db*/*db* mouse model, or it may indicate that Treg modulation alone is insufficient to reverse the metabolic aspects of diabetes.

**Figure 2:**
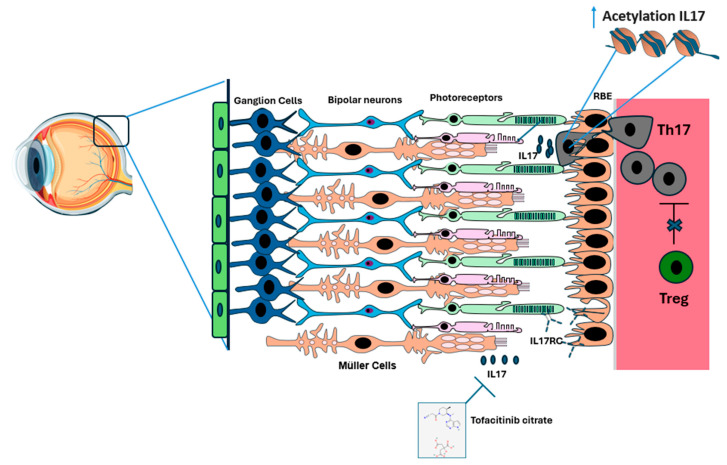
Infiltration of Th17 Cells into the blood–retina barrier (BRB) in diabetic retinopathy (DR). In a healthy eye, the retinal layers, comprising epithelial cells, ganglion cells, bipolar cells, rods, and cones, along with the retinal pigment epithelium (RPE), Bruch’s membrane, and choroid, remain intact. We hypothesize that as diabetes mellitus progresses, Tregs (in green) lose their ability to effectively suppress Th17 cells (in gray). As a result, Th17 cells infiltrate the RPE. This is followed by increased expression of IL17 through increased acetylation of IL17 in Th17 cells and possibly in Müller cells. When IL17 binds to its receptor IL-17RC on the surface of photoreceptors and endothelial cells, the Fas mechanism is activated, leading to photoreceptor and endothelial cell apoptosis. Tofacitinib citrate appears to inhibit IL17 function and reduce its associated inflammation.

We could hypothesize that DR may be driven by an imbalance in the Th17/Treg axis, similar to what is observed in type 2 diabetes (T2D) [85]. This imbalance involves a reduction in Treg cells and an increase in Th17 infiltration into the retina, leading to a pro-inflammatory environment that promotes DR progression. Mechanistically, the breakdown of the BRB may facilitate Th17 cell infiltration. However, this would suggest that Treg infiltration should be comparable to Th17 infiltration, which contradicts our current observations. Alternatively, Treg cells may be unable to effectively suppress inflammation in DR due to elevated insulin levels, a hallmark of T2D [85,86]. Additionally, the increased Th17 cell infiltration could be linked to their specific ability to be recruited by certain chemokines and the support of PECAM1 [87,88,89]. This unique recruitment ability of Th17 cells contributes to a cycle of retinal vascular leakage, hemorrhages, and ultimately, vision impairment (Figure 2).

## 4. Treatment Alternatives

Focusing on therapeutic strategies that restore the balance between Tregs and Th17 cells may offer new avenues for treating DR by controlling inflammation and preventing further retinal damage. For example, enhancing the function of Tregs or inhibiting the pro-inflammatory activity of Th17 cells may help mitigate the chronic inflammation that drives DR. Targeting the molecular pathways that regulate Treg and Th17 differentiation and function could provide novel strategies for managing DR and other autoimmune and inflammatory diseases [90,91].

### 4.1. Natural Compounds

**Figure 3:**
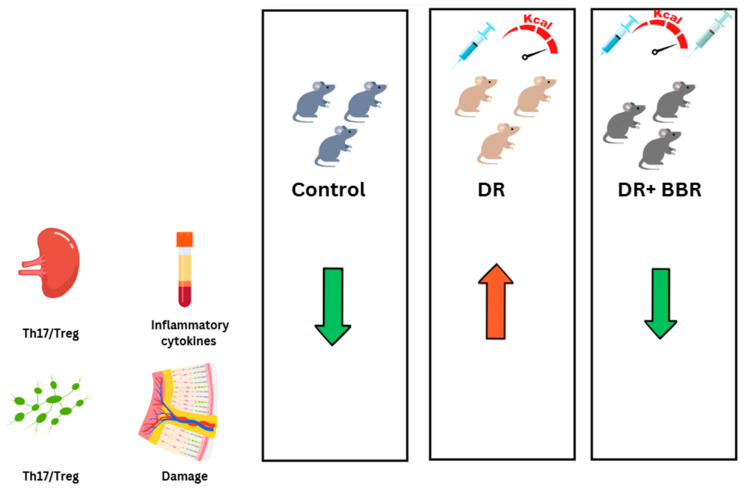
Berberine (BBR) is a potential therapy for diabetic retinopathy (DR) through inflammation reduction. C57BL/6 mice were treated with streptozotocin (STZ) to induce diabetes and then fed a high-fat diet, with or without a 5-week BBR treatment. The results published by Yang et al. indicate that BBR reduced the Th17/Treg ratio in the spleen and lymph nodes, decreased inflammatory cytokine expression, and mitigated retinal damage in DR mice.

Interestingly, natural compounds like berberine appear to significantly mitigate the detrimental role of Th17 in DR. An interesting study by Yang et al. explored the potential therapeutic effects of berberine (BBR) on DR, specifically focusing on its impact on the Th17/Treg cell balance [92]. In this study, a streptozotocin (STZ)-induced diabetic mouse model was used, with BBR treatment administered over a period of 5 weeks. The results revealed that BBR alleviated retinal damage, reduced inflammatory cytokines (TNF-α, IL-1β, IL-6), and favorably altered immune cell populations. Specifically, BBR inhibited the Th17 cells while promoting Treg cell expansion in the spleen and lymph nodes, thus reducing the Th17/Treg ratio (Figure 3). In vitro studies further demonstrated that BBR directly down-regulated the Th17/Treg ratio by modulating key transcription factors RORγt and Foxp3 in T cells and indirectly by influencing dendritic cells to enhance anti-inflammatory cytokine production (TGF-β, IDO) and reduce pro-inflammatory cytokine secretion. These findings suggest that BBR exerts its therapeutic effects in DR by modulating the immune response through the regulation of the Th17/Treg axis, offering a promising treatment strategy for DR [92]. Additionally, curcumin, which is known for its histone acetyltransferase inhibitory properties, has been shown to effectively suppress the high glucose-enhanced histone acetylation of IL17A-targeted genes in DR [75].

### 4.2. Chemokines Modulation

Neutralizing IL17A offers a promising therapeutic strategy for diabetic retinopathy (DR). Neutralizing IL17A has been shown to reduce both retinal vascular permeability and pathological neovascularization by disrupting its pro-inflammatory and proangiogenic effects [93,94,95]. Moreover, IL17A inhibition decreases endoplasmic reticulum (ER) stress, which is intricately linked to IL17A in a positive feedback loop. This dual action not only diminishes vascular damage but also interrupts key pathways, such as the TXNIP/NLRP3 axis, involved in the progression of DR. These findings suggest that IL17A neutralization could directly address the inflammatory and vascular components of DR, providing a targeted approach for managing this vision-threatening complication [93]. Further evidence suggests that targeting the IL23–Th17–IL17A pathway may reduce complications in patients with DR. In STZ-induced diabetic retinopathy in rats, IL17A+CD4+ T cell levels, as well as IL17A mRNA and protein expression, were significantly elevated in the peripheral blood and retina. Intravitreal injections of an anti-IL-23Rp19 antibody effectively reduced IL17A levels, improved blood–retinal barrier integrity, and decreased retinal micrangium and endothelial cells. These findings highlight the therapeutic potential of targeting the Th17/IL-23/IL-17A axis to mitigate DR progression [96]. Furthermore, Irisin, a hormone produced primarily by skeletal muscles in response to physical activity, has been shown to possess anti-inflammatory properties and was used to investigate its potential protective effects against DR. The research found that irisin levels were significantly lower in T2DM patients, and its correlation with IL-17A suggested that irisin may help mitigate IL-17A-driven inflammation in NPDR, potentially offering a therapeutic target for DR management (Table 1) [97].

### 4.3. Immunomodulatory Drugs

Supporting Treg function, either through direct transfer of healthy Tregs or indirectly by promoting cytokines typically produced by Tregs, is emerging as a potential treatment approach. For example, a study on PDR found that IL35, an anti-inflammatory cytokine, plays a protective role by inhibiting Th17 cell differentiation and IL17 expression. In PDR patients, IL35 levels were reduced while IL17 levels were elevated, and an increase in Th17 cell frequency was observed. However, IL35 treatment decreased Th17 cell frequency and IL17 production by reducing the key transcription factors (ROR α and RORγt) necessary for Th17 differentiation. These findings suggest that IL-35 could help to provide protection against PDR by modulating the Th17/IL17 pathway [98]. In an intriguing study, Robinson et al. explored the relationship between IL6 trans-signaling and oxidative stress in DR. Using a streptozotocin (STZ)-induced mouse model of early DR, they demonstrated that blocking IL6 trans-signaling with sgp130Fc treatment significantly reduced oxidative stress in retinal endothelial cells. The results showed a restoration of antioxidant capacity and a reduction in markers of oxidative damage, such as superoxide levels and lipid peroxidation, in both the retina and the systemic circulation. These findings further highlight the role of IL-6 trans-signaling in mediating diabetes-induced oxidative damage and suggest that its inhibition could mitigate oxidative stress in DR [99]. As IL6 is essential for Th17 differentiation, we can speculate that targeting IL6 could potentially lead to a reduction in the number of Th17 cells infiltrating the retina. Directly targeting Th17 could be another viable option to reduce inflammation in DR by modulating the balance between Th17 and Tregs. Inhibition of RORγ has been shown to decrease the abundance of pro-inflammatory Th17 cells while enhancing the functionality of Tregs. In animal models, treatment with the RORγ inhibitor SR2211 reduced retinal vascular leakage, VEGF protein levels, and inflammatory factors such as TNF and ICAM-1. This suggests that RORγ inhibition could help attenuate retinal inflammation and vascular damage, providing a potential therapeutic strategy for DR [100]. Finally, the plasticity of the Th17/Treg axis, particularly the ability to convert pathogenic Th17 cells into less inflammatory types, remains a potential therapeutic option that has yet to be investigated in the context of DR.

An investigation of the clinical trials database (https://clinicaltrials.gov/) revealed that while numerous trials have aimed to reduce diabetic retinopathy (DR) symptoms by targeting vascular regeneration, lipid metabolism, or neural growth, no reported attempts have focused on the Th17/Treg axis. Additionally, although extensive cytokine profiling in DR patients has indicated a pro-inflammatory profile, including IL-17A and IL-1B, these cytokines have yet to be established as regular diagnostic markers for DR development. Given the growing recognition of immune dysregulation in DR pathophysiology, we anticipate that research efforts in the coming years will direct increasing amounts of focus towards exploring the Th17/Treg balance and their associated cytokines as potential biomarkers and therapeutic targets. This shift could open up new avenues for immune-modulating strategies to prevent or slow DR progression, complementing existing vascular and metabolic approaches.

## 5. Conclusions

Diabetic retinopathy remains a leading cause of vision loss, with its progression intricately tied to hyperglycemia-induced vascular damage and immune system dysregulation. The imbalance between Th17 and Treg cells plays a pivotal role in driving the inflammatory processes central to DR. Therapeutic approaches aimed at restoring the Th17/Treg balance, whether through cytokine modulation or Treg cell augmentation, offer promising avenues for mitigating retinal damage and preserving vision. Further research into the molecular mechanisms of this axis is essential to develop effective, targeted treatments that address both the inflammatory and vascular components of DR. By utilizing these strategies, improved patient outcomes and innovative therapies become increasingly attainable.

## Figures and Tables

**Table 1:** Current therapeutic strategies targeting Th17/Treg axis in DR.

Therapeutic Strategy	Mechanism of Action	Key Findings and Research Status	Ref.
**Natural Compounds**			
Berberine (BBR)	Inhibits Th17 and promotes Tregs. Reduces inflammatory cytokines (TNF-α, IL-1β, IL-6). Downregulates RORγt and upregulates Foxp3. Enhances anti-inflammatory cytokines (TGF-β, IDO).	In STZ-induced diabetic mice, BBR reduced retinal damage and inflammation, offering a promising strategy for DR treatment.	[92]
Curcumin	Inhibits histone acetyltransferase activity, reducing IL-17A expression.	Suppresses glucose-induced histone acetylation of IL17A-targeted genes in DR.	[75]
**Chemokine Modulation**			
IL-17A Neutralization	Reduces retinal vascular permeability and neovascularization by inhibiting inflammation and ER stress. Disrupts the TXNIP/NLRP3 axis.	IL-17A blockade reduced vascular damage and inflammation in DR models.	[93,94,95]
Irisin Therapy	Skeletal muscle-derived hormone reduces IL-17A-driven inflammation.	Lower irisin levels in T2DM patients correlated with higher IL-17A, suggesting potential protective effects in NPDR.	[97]
**Immunomodulatory Drugs**			
IL-35 Therapy	Inhibits Th17 differentiation, reduces IL-17 expression, and suppresses RORα and RORγt transcription factors.	In PDR patients, IL-35 levels were reduced while IL-17 and Th17 cell frequencies were elevated. IL-35 treatment reversed these effects.	[98]
IL-6 Trans-Signaling Blockade	Blocks IL-6-mediated Th17 differentiation and oxidative stress in DR. Restores antioxidant capacity and reduces oxidative damage (superoxide levels, lipid peroxidation).	In STZ-induced DR models, IL-6 inhibition reduced retinal oxidative stress, suggesting its potential role in preventing Th17-mediated inflammation.	[99]
RORγ Inhibition (SR2211)	Suppresses Th17 differentiation while enhancing Treg function. Reduces VEGF, TNF, and ICAM-1 levels.	In animal models, SR2211 reduced retinal vascular leakage and inflammation, offering a new therapeutic approach.	[100]
Th17 to Treg Conversion	Explores the potential of converting pathogenic Th17 cells into regulatory T cells.	Still an unexplored area in DR treatment, but holds promise for future investigations.	

## Data Availability

Not applicable.
